# Drought Drives Spatial Variation in the Millet Root Microbiome

**DOI:** 10.3389/fpls.2020.00599

**Published:** 2020-05-28

**Authors:** Tuesday Simmons, Alexander B. Styer, Grady Pierroz, Antonio Pedro Gonçalves, Ramji Pasricha, Amrita B. Hazra, Patricia Bubner, Devin Coleman-Derr

**Affiliations:** ^1^Department of Plant & Microbial Biology, University of California, Berkeley, Berkeley, CA, United States; ^2^Plant Gene Expression Center, United States Department of Agriculture–Agriculture Research Service, Albany, CA, United States

**Keywords:** plant microbiome, abiotic stress, root endosphere, drought, plant microbe interaction

## Abstract

Efforts to boost crop yield and meet global food demands while striving to reach sustainability goals are hindered by the increasingly severe impacts of abiotic stress, such as drought. One strategy for alleviating drought stress in crops is to utilize root-associated bacteria, yet knowledge concerning the relationship between plant hosts and their microbiomes during drought remain under-studied. One broad pattern that has recently been reported in a variety of monocot and dicot species from both native and agricultural environments, is the enrichment of Actinobacteria within the drought-stressed root microbiome. In order to better understand the causes of this phenomenon, we performed a series of experiments in millet plants to explore the roles of drought severity, drought localization, and root development in provoking *Actinobacteria* enrichment within the root endosphere. Through 16S rRNA amplicon-based sequencing, we demonstrate that the degree of drought is correlated with levels of *Actinobacterial* enrichment in four species of millet. Additionally, we demonstrate that the observed drought-induced enrichment of *Actinobacteria* occurs along the length of the root, but the response is localized to portions of the root experiencing drought. Finally, we demonstrate that *Actinobacteria* are depleted in the dead root tissue of Japanese millet, suggesting saprophytic activity is not the main cause of observed shifts in drought-treated root microbiome structure. Collectively, these results help narrow the list of potential causes of drought-induced *Actinobacterial* enrichment in plant roots by showing that enrichment is dependent upon localized drought responses but not root developmental stage or root death.

## Introduction

Drought is a major obstacle to agricultural productivity. It is currently the climate phenomenon with the greatest negative impact on cereal production (Lesk et al., 2016), and the severity and frequency of drought is expected to increase in the coming decade (Vicente-Serrano and Lopez-Moreno, 2014; Spinoni et al., 2018). As such, it represents one of the largest challenges to food security (Kogan et al., 2019), especially considering the anticipated increases in food production that will be needed to feed the growing world population (Ray et al., 2013). Historically, crop breeding has helped select for drought resistant cultivars, but such efforts are often time and labor intensive (Coleman-Derr and Tringe, 2014). For these reasons, development of alternative strategies of protection against drought’s negative impacts on crop fitness are needed (Kang et al., 2009; Lesk et al., 2016).

Microbially mediated crop fortification is currently touted as an attractive strategy for mitigating drought stress (Naylor and Coleman-Derr, 2018). Additionally, it has been shown that plant growth promoting microorganisms (PGPM) have a greater effect on plant growth during drought compared to well-watered conditions (Rubin et al., 2017), and it is well established that crops grown in arid desert ecosystems act as “resource islands” for cultivating known PGPM in the surrounding soil (Köberl et al., 2011; Marasco et al., 2012). Recent work has demonstrated that drought has a strong impact on the structure and activity of the root microbiome, and is correlated with a significant enrichment in lineages of monoderm bacteria within the root and rhizosphere that is not observed in the surrounding soil (Naylor et al., 2017; Santos-Medellín et al., 2017; Edwards et al., 2018; Fitzpatrick et al., 2018; Xu et al., 2018). It should be noted that within these studies, those that investigated single host species (Santos-Medellín et al., 2017; Edwards et al., 2018; Xu et al., 2018) reported an enrichment of primarily *Actinobacteria*, *Firmicutes* to a lesser degree, and some lineages of *Chloroflexi*. Other studies that looked at multiple host species (Naylor et al., 2017; Fitzpatrick et al., 2018) reported that *Actinobacteria* were the enriched taxa across hosts, and additional studies have noted that *Actinobacteria* dominate portions of the root microbiome for desert-adapted plants (Marasco et al., 2018). For this reason, the primary focus of this research is on understanding the causes of the *Actinobacterial* enrichment in the endosphere.

It has been shown that applications of *Actinobacteria*, in particular *Streptomyces spp.*, may benefit host fitness under drought (Yandigeri et al., 2012; Xu et al., 2018); many strains are antagonistic toward pathogens (Millard and Taylor, 1927; Newitt et al., 2019; Suárez-Moreno et al., 2019), produce beneficial secondary metabolites, and assist in nutrient acquisition (Sathya et al., 2017). However, the spatial-temporal dynamics of drought-induced enrichment of *Actinobacteria* remains largely uncharacterized, and it is unclear if this restructuring occurs in all roots – and all parts of each root – within the root system. Water availability is known to vary within the root zone, both at the macro scale (due to the falling water table) and the micro scale (due to the heterogeneous nature of soil composition) (D’odorico and Porporato, 2006). Whether the resulting variability in the degree of water stress that is likely to occur across a drought-stressed root system corresponds with differential recruitment of microbes is also currently unknown.

A better understanding of the underlying spatial organization of the observed *Actinobacterial* enrichment may help identify the underlying causes of this phenomenon. At present, it is unknown if the enrichment is driven by local or systemic changes in host physiology or metabolism. If the drought-induced shifts in the root microbiome are limited to roots that directly perceive a lack of water, then localized responses to drought stress could serve as a signal for *Actinobacteria* enrichment. For example, perhaps root tissue death (Liu et al., 2009) triggers the proliferation of saprophytic lineages within *Actinobacteria*. Alternatively, if the observed enrichment also occurs in the relatively few roots of drought stressed plants with access to water, this phenomenon may instead be driven by systemic processes, such as above-ground, vasculature-mediated changes in plant metabolism that are translocated throughout all root tissue.

In addition, physiological and functional properties of root tissue differ along the root’s longitudinal axis even within the context of a single root (Petricka et al., 2012). Older root tissue closer to the stem is responsible for root hair and lateral root development, while the youngest tissue at the tip is responsible for active growth, cell division, and is the site of the majority of root exudation (Canarini et al., 2019). Whether the drought-induced enrichment in *Actinobacteria* occurs across the entirety of an individual root’s length, or is specific to older or younger tissue types, is currently unknown.

To address these knowledge gaps, we have conducted a series of field and greenhouse-based experiments to allow for spatially resolved measurements of the compositional shifts within the millet root microbiome that occur in response to drought. Millets are a polyphyletic group of cereal crops that provide a primary source of food and fodder for hundreds of millions of people in the dry regions of Africa and Asia (Patil, 2017). They are often grown on marginal lands where irrigation is rain fed and sporadic, and as such are among the crops most exposed to water stress during periods of drought (Kumar et al., 2018). In this study, we worked with five different members of the Paniceae tribe: *Setaria italica* (foxtail millet), *Pennisetum glaucum* (pearl millet), *Panicum miliaceum* (proso millet), and *Echinochloa esculenta* (Japanese barnyard millet), which are all millets, and *Sorghum bicolor*, a related cereal crop. We set out to test whether millets, like other cereal crops, are enriched with *Actinobacteria* when drought stressed, and whether this enrichment is correlated with the severity of drought. We also tested whether this pattern is specific to a particular root tissue age, and if enrichment occurs at similar levels from the actively growing root tip to older and more mature root tissue basal to the stem. This would demonstrate whether recently reported drought-induced changes in the plant root microbiome are driven by root specific factors that are independent of the root tissue’s developmental stage. Additionally, using a split-pot experimental design, we test whether observed enrichment of *Actinobacteria* is localized to drought-stressed roots, or systemically throughout the root system. Finally, we investigate localized root death as a potential primary driver of the observed bacterial community shifts.

## Materials and Methods

### Drought Gradient and Multi-Species Field Design

Four species of millet – all members of the *Paniceae* tribe – were planted on May 19th, 2015 at the University of California at Berkeley’s Gill Tract research field in Albany, California (37°53′12.3″N 122°18′00.3″W): *Setaria italica* (foxtail millet), *Pennisetum glaucum* (pearl millet), *Panicum miliaceum* (proso millet), and *Echinochloa esculenta* (Japanese millet). Seeds were planted directly in the field with 8–10 seeds per hill and hills 25–30 cm apart. The four species were subjected to three different watering regimes: control (watered on the day of planting then weekly until maturity), moderate drought (watered on the day of planting, weekly for the next 5 weeks, and water then withheld until maturity), and severe drought (watered on the day of planting, once the following week, then withheld until maturity). Watering treatments were applied for 6 h using drip irrigation tape with 1.89 L/h rate flow emitters. Tissue and soil samples were harvested 24 weeks post-germination, after each species had reached maturity. Root systems for each species are structurally similar; they all are fibrous and lack a tap root, typical of monocotyledons. Bulk soil samples were taken 30 cm from the base of the plant at the same time point; root/rhizosphere were collected as detailed in Simmons et al. (2018) and stored in phosphate buffer at −80°C until further processing.

### Sub-Sectioned Root Field Experimental Design

*Sorghum bicolor* was chosen for this experiment due to its larger root structure, which allowed for increased precision during root system dissection. *S. bicolor* seeds were planted on June 21, 2017 at the USDA Gill Tract research field in Albany, California under a sheet of plastic mulch to reduce weed growth. Plants were watered weekly for the first 3 weeks after planting. For each application, water was administered for 6 h using drip irrigation tape with 1.89 L/h rate flow emitters. Samples were taken for whole root systems and three single roots after 1 week. After 2, 3, 9, and 11 weeks, we collected whole root systems and six single roots, three of which were further partitioned into 3 approximately equal length subsections. At each time point, a single bulk soil sample was collected for each plant, approximately 30 cm from the base of the plant. When collecting root samples, the single roots were collected first, and the remaining roots were pooled and considered to be the whole root system (Supplementary Figure S1). The single roots were selected from the system by: presence of root tip and minimal lateral root growth. After sample collection, roots were placed into sterile conical tubes with phosphate buffer and stored at −20°C until further processing.

### Split-Pot Experimental Design

Fifteen *E. esculenta* seeds were planted in sterile pots filled with sifted field soil and grown for 2 weeks before transferring 12 plants to a split-pot design (Supplementary Figure S2). The split-pot design consisted of two 1-L square sterile pots connected together with adhesive and filled with field soil pre-sifted through a 1 cm sieve; transplanted seedling roots were partitioned such that half of the root system was located on each side of the split-pot system. After a 1 week acclimation period, three different watering regimes were initiated: full water (W; water was applied on both sides), full drought (D; drought was applied on both sides), and half-water/half-drought (W/D; water was applied only on one side) with four plants per treatment. A plastic sheath was applied to the outside of the pot on the drought side of the W/D plants to prevent water from moving up through the base of the pot from the water reservoir below. Plants were grown for an additional 10 days before collecting bulk soil from both sides of the pot; root/rhizosphere samples were collected as described above and stored in phosphate buffer at −20°C until further processing.

### Live-Dead Root Community Profiling Design

Five *E. esculenta* seeds were planted per pot in 0.25L sterilized pots (13) in a greenhouse. After 1 week, the pots were thinned to one plant each, and 1 week later the plants were transplanted to sterile 4 L pots filled with sifted (1 cm sieve) field soil. They were grown for an additional week before initiating drought stress on half of the plants (28 days post-germination). One day after the start of drought treatment, a subsection of roots was severed from the rest of the plant by connecting a razor blade to the end of a wooden stake and pushing it at a 45° angle through the root zone, starting at the base of the plant (Supplementary Figure S3). The blade was then removed, and the wooden stake replaced within the soil to identify the location of separated tissue. After 10 days of drought, root and rhizosphere samples were collected from both living and dead roots and placed into conical tubes with phosphate buffer. Samples were stored at −20°C until further processing. Additional severed and live root samples were collected from replicate plants to perform cell viability assays on the roots. These assays were performed on roots collected on the day of root detachment, 3 days later, and on the day samples were collected for community profiling (9 days post-detachment). To assay cell death, we used the Plant Cell Viability Assay kit (Sigma-Aldrich, Darmstadt, Germany) according to the manufacturer’s instructions.

### Root/Rhizosphere Processing and DNA Extraction

The methods used here are modifications of what is described in Simmons et al. (2018). Roots frozen in phosphate buffer solution were thawed at 4°C and washed by sonication in a Bioruptor Plus ultrasonicator (Diagenode, Denville, NJ, United States) at 4°C for 10 min. Roots were removed from vials and rinsed twice with autoclaved water. For each plant in the subsectioning experiment, three of the clean individual roots were then cut into three sections of equal length. Roots not being processed immediately were placed in fresh sterile phosphate buffer and frozen at −80°C. Rhizosphere soil samples from the sonicated vials were centrifuged (10 min at 4 °C, 4,000 × *g*), and DNA was extracted by processing approximately 250 mg of each sample with MoBio’s PowerSoil kit (prior to Qiagen purchasing MoBio). DNA was extracted from root samples by grinding to a powder with liquid nitrogen, mixing 600–700 mg powder with CTAB buffer, and washing with phenol chloroform-isoamyl alcohol. For individual and sectioned roots in the subsectioning experiment, DNA was extracted using approximately 50 mg of tissue in MoBio’s PowerPlant kit. Bulk soil DNA was extracted with MoBio’s PowerSoil kit.

### 16S Amplification and Sequencing

All samples were amplified in triplicate using barcoded universal primers (180 s at 98 °C, 30 cycles of: 98 °C for 45 s, 78°C for 10 s, 55°C for 60 s, and 72°C for 90 s, then 600 s at 72°C followed by a 4°C hold) for the v3-v4 region (341 F, 5′-CCTACGGGNBGCASCAG-3′ and 785 R, 5′-GACTACNVGGGTATCTAATCC-3′) of the 16S rRNA gene according to Simmons et al. (2018). Additionally, PNAs matching chloroplast and mitochondrial 16S sequences were spiked into PCRs (2.28 μM final concentration) to prevent amplification of these unwanted reads. Replicate PCR products were pooled and quantified using Qubit HS assay; 100 ng from each sample was pooled together and cleaned using AMPureXP magnetic beads before a final quantification and dilution to 10 nM for sequencing at the UC Berkeley Vincent Coates Sequence Facility via Illumina MiSeq (v3 chemistry, 300 bp paired-end sequencing). Reads were demultiplexed in QIIME2 (Bolyen et al., 2018) and then passed to DADA2 (Callahan et al., 2016) where sequences were trimmed to ensure minimum median Phred Q-scores of 30 or greater at any given base pair position prior to denoising and Amplicon Sequence Variant (ASV) inference; 500,000 reads were used to train error-rate models, but otherwise all other pipeline default settings were used. A taxonomy classifier was trained to the V3-V4 region of sequences from the August 2013 version of GreenGenes 16S rRNA gene database via Naive Bayesian methods in QIIME2 and used to assign taxonomic associations to ASVs. All subsequent statistical analyses were completed in R; scripts and datasets can be found at https://github.com/colemanderr-lab. The phylogenetic tree of indicator species was generated using the online tool: Interactive Tree Of Life (iTOL) v5 (Letunic and Bork, 2019). All raw reads are deposited in the NCBI Short Read Archive at accession PRJNA607579.

## Results

### Bacterial Root Microbiome Is Driven by Host Species and Degree of Drought

Recent work has shown that drought leads to enrichment of *Actinobacteria* within the root microbiome of a wide variety of angiosperms, including many cereal crops (Naylor et al., 2017; Fitzpatrick et al., 2018). To establish whether drought produced similar enrichment patterns in millets, as well as explore whether such enrichments are correlated with the severity of drought treatment, we conducted a field experiment in which four millet species (see section “Materials and Methods”) were subjected to three different watering regimes (control, moderate drought, or severe drought) in a field with acidic silty loam soil (pH 5.2) (Naylor et al., 2017). At the time of sample collection (164 days post-germination), gravimetric soil moisture content was found to be significantly different (*p* = 1.56E-17, one-way ANOVA) between all three treatments:16.1% for control (*n* = 12, SD = 2.89%), 5.5% for moderate drought (*n* = 12, SD = 1.27%), and 3.7% for severe drought (*n* = 12, SD = 0.67%). Aboveground phenotypes measured at root collection demonstrate that despite millet’s drought tolerance, drought treatment had a significantly negative impact on plant growth (Supplementary Figures S4–S6). Plant height was negatively impacted by drought stress across three millet species (phenotypic data for one species was not collected; Wilcoxon rank-sum test, *p* < 0.001), with the greatest impact observed under severe drought stress. Additionally, one variety (*E. esculenta*) displayed a significant reduction in grain ear length (Wilcoxon rank-sum test, *p* < 0.01) during severe drought, and median values of ear length decreased with increasing drought severity across all three species. Together these results suggest that drought treatment negatively impacted millet fitness, and that the degree of impact was correlated with drought severity.

**FIGURE 1:**
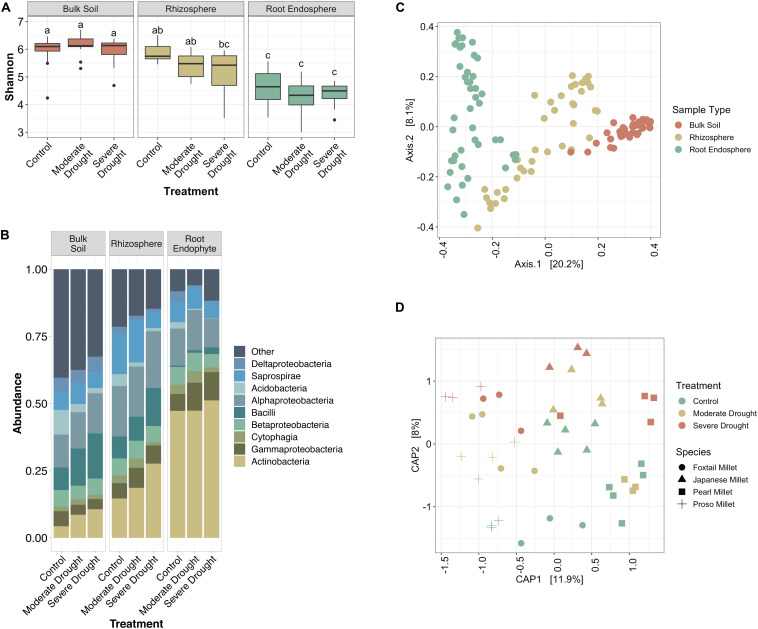
Millet root microbiome profiles vary by severity of drought. **(A)** Box-and-whiskers plot of Shannon’s diversity of bulk soil, rhizosphere, and root endophyte communities subjected to a gradient of water conditions (control, moderate drought, and severe drought). Letters indicate significantly different groups (*p* < 0.05, Tukey’s test) From left to right sample size: *n* = 13, 13, 13, 12, 12, 15, 16, 15, 13. **(B)** Relative abundance of the top 9 most abundant bacterial classes in each compartment of the root microbiome. **(C)** Ordination plot (PCoA) of all samples based on Bray-Curtis dissimilarity, colored by the source material. **(D)** Constrained ordination plot (CAP) of root endophyte samples based on Bray-Curtis dissimilarity. Colors indicate treatment type and shape indicates host species.

To investigate how bacterial communities shifted during increasing levels of drought stress in millet, we profiled the soil, rhizosphere, and root endophyte communities by barcoded amplicon sequencing. We observed that while there is a significant difference between the alpha diversity in bulk soil, rhizosphere, and endosphere samples (*p* < 0.05, Tukey’s test), there is not a significant difference between the drought treatments within the same sample type (Figure 1A). It is also noteworthy that while the root endosphere communities are less diverse than their corresponding rhizospheres in the control and moderate drought conditions, this is not the case for severe drought (Figure 1A). Additionally, drought provoked a relative increase in *Actinobacteria* within root endophyte, rhizosphere, and unexpectedly, bulk soil communities (Figure 1B). Moderate drought, which was initiated later in plant development, failed to provoke a strong enrichment in *Actinobacteria* within roots or rhizosphere, a result that is consistent with recent research that demonstrated that drought occurring earlier in development provokes a more substantial shift in *Actinobacteria* (Xu et al., 2018).

To explore how bacterial community composition varied across both host species and treatment, PERMANOVA and ordination analyses were performed on Bray Curtis distances. These analyses revealed significant differences in composition across the dataset are driven by primarily by sample type (F-statistic = 17.864, *p* < 0.001), with weaker effects contributed by host species (F-statistic = 4.952, *p* < 0.001), and watering treatment (F-statistic = 3.989, *p* < 0.001); the strong clustering by sample type is confirmed by Principle Coordinate Analysis (Figure 1C). When considering root endophyte communities alone, the percent of variance attributable to water treatment is 9.6% (*p* < 0.001), and the percent of variance attributable to host species is 21.2% (*p* < 0.001), and Constrained Analysis of Principle Coordinates reveals clustering by both species and treatment (Figure 1D). Taken together, these results demonstrate that the millet root microbiome responds to drought treatment in a manner similar to other previously reported plant systems, making them suitable systems for the experiments described below.

### Actinobacteria Enrichment Pattern Occurs Along the Length of the Root

**FIGURE 2:**
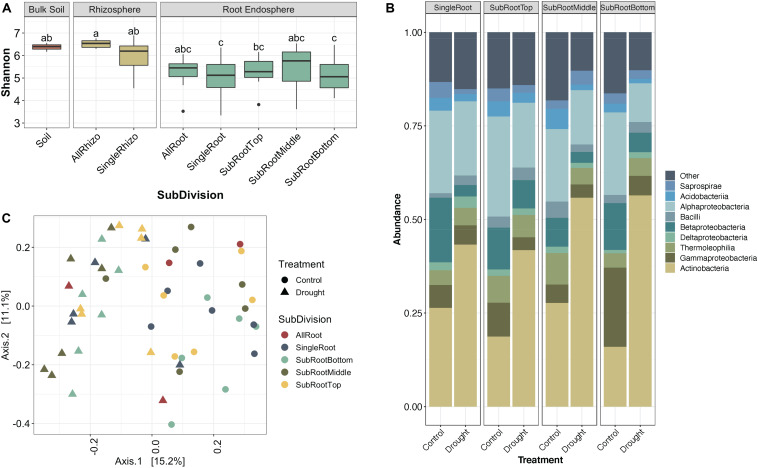
Variation in sorghum root microbiome communities by root age. **(A)** Box-and-whiskers plot of Shannon’s diversity of bulk soil, rhizosphere, and root endophyte communities separated by root age. SubDivision (x-axis) indicates whether samples came from whole root systems (AllRoot; the bulk of roots remaining after six individual roots were collected), individual roots (SingleRoot), or subsectioned roots (SubRootTop is closest to the plant and SubRootBottom is the root tip). Letters indicate significantly different groups (*p* < 0.05, Tukey’s test). **(B)** Relative abundance of the top 9 most abundant bacterial classes in the root endosphere separated by water treatment. **(C)** PCoA plot of root samples based on Bray-Curtis dissimilarity, colored by root age where open circles are well-watered control roots and triangles are drought-stressed roots.

**FIGURE 3:**
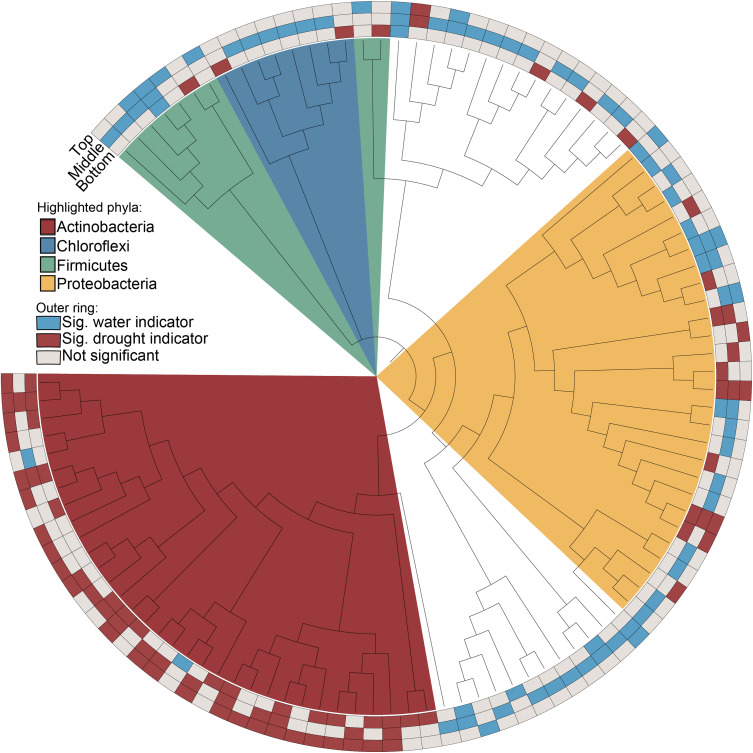
Indicators of drought and watered conditions by root subsection (root tip, middle, or base). Nodes represent genera where taxonomic information was available to group ASVs by this rank. Red and blue boxes indicate nodes that are significant indicators of drought and water conditions after Dufrene-Legendre analysis, respectively (*p* < 0.05, indcls >0.5). Nodes are highlighted by phyla [*Actinobacteria* (red), *Chloroflexi* (blue), *Firmicutes* (green), *Proteobacteria* (yellow)].

We hypothesized that enrichment of *Actinobacteria* would be observable throughout the root system rather than in specific root zones or types. After profiling the bacterial communities at sub-root system spatial resolution (Supplementary Figure S1), we found that an enrichment of *Actinobacteria* under drought treatment was observed within single roots and across all three subsections of an individual root, with concomitant decreases in most *Proteobacterial* classes (Figure 2). Additionally, *Actinobacteria* are the predominant indicator taxa of drought within each subsection according to Dufrene-Legendre indicator species analysis (Figure 3; Dufrêne and Legendre, 1997). This demonstrates that *Actinobacterial* enrichment is not unique to the actively growing root tip where most new microbial recruitment to the root endosphere is thought to occur (Shyam et al., 2017). Notably, however, both *Firmicutes* and *Chloroflexi* appeared more often as indicators of the watered condition, in contrast to what has been observed in several other studies.

Additionally, as part of this experiment, a comparison of intrareplicate and intraplant variation within root sample types was conducted. We hypothesized that due to the stochastic nature of root colonization events and founder effects at smaller physical scales, variation between replicates would be greater in subsections rather than whole root systems. As expected, we observed that as the spatial resolution increases from whole root systems toward individual root subsections, variation between sample replicates increases (Supplementary Figure S7). Additionally, we observed that root communities of replicates from the same plant are more similar to each other than replicates from different plants (F-statistic = 507.4, *p* < 0.0001, Supplementary Figure S8) and replicates from the same root are again more similar compared to replicates from different roots of the same plant (F-statistic = 7.453, *p* < 0.007, Supplementary Figure S8). Interestingly, root tips account for greater dissimilarity when comparing subsections of roots both within and between plants, likely indicating that root tips are sites of stochastic colonization while older middle and basal sections of roots have communities stabilized through selection and competition (Supplementary Figure S8).

### Localized Drought Causes Enrichment of Actinobacteria

**FIGURE 4:**
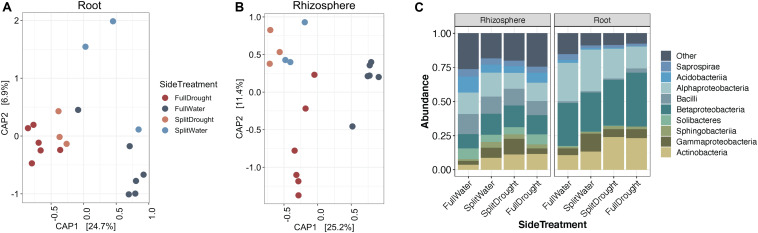
Effect of a split-pot watering system on the root microbiome. **(A)** CAP plot of root endosphere samples colored by water treatment in Japanese millet. **(B)** CAP plot of rhizosphere samples colored by water treatment in Japanese millet. **(C)** Relative abundance of the top 9 most abundant bacterial classes in either the rhizosphere or endosphere of the different watering treatments of Japanese millet.

While the enrichment of *Actinobacteria* bacteria during drought does not appear to depend on the developmental stage of root tissue, it remains unclear whether this enrichment is driven by localized processes at the site of drought, or by systemic responses affecting the entire root system. Using Japanese millet grown in a split-irrigation design (Supplementary Figure S2), Constrained Analysis of Principal Components (CAP) of amplicon-based bacterial community profiling of the roots and rhizosphere revealed that root endophyte communities from the watered side of the were more similar to the communities of fully watered plants, while the drought-treated side of the split-irrigation plants were more similar to communities found in fully drought-treated plants (Figures 4A,B). Comparisons of the root endophyte community relative abundance patterns between the two sides of split-irrigation plants and between their fully watered and drought treated counterparts demonstrated that there is an increase in the abundance of *Actinobacteria* in drought-treated roots in both full drought and split-drought treatments (Figure 4C). Collectively, these results suggest that *Actinobacterial* enrichment occurs locally at the site of drought induction rather than systemically.

### Root Death Does Not Drive Enrichment of Actinobacteria

**FIGURE 5:**
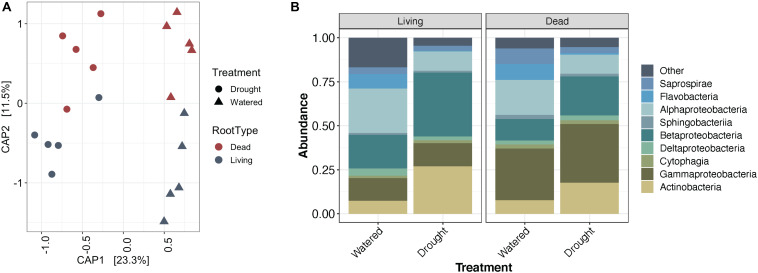
Impact of root death on Japanese millet root endophyte communities. **(A)** CAP plot of root endosphere samples colored by root type and shaped by water treatment. **(B)** Relative abundance of the top 9 most abundant bacterial classes in the root endosphere separated by root type and water treatment.

A subset of *Actinobacteria* lineages can exist as saprophytes (Barka et al., 2016), deriving their carbon from dead and decaying plant material. As localized root tissue death can accompany severe drought stress (Liu et al., 2009), we surmised that the observed local enrichment in *Actinobacteria* could be driven by root death. To test this hypothesis, we induced localized root death through mechanical severing and compared levels of *Actinobacteria* recruitment across the root system under drought stress and induced root death treatments (Supplementary Figure S3). To confirm root death, we used a live-dead stain to test for cell viability. A subset of cells remained viable for 3 days following root separation, but by 9 days cells within separated roots were no longer viable (Supplementary Figure S9). After community profiling of the root (Figure 5) and rhizosphere (Supplementary Figure S10) fractions, we observed that in addition to the expected differences in bacterial community composition between drought-treated and watered samples, communities in living or dead tissue showed significant differences. Performing PERMANOVA on the root endophyte samples showed that water treatment explains 23.2% of variance in beta-diversity (*p* < 0.001), and tissue death explained 11.6% (*p* = 0.003).

Contrary to our hypothesis, an enrichment of *Actinobacteria* was not observed in dead roots compared to living roots under either watering condition (Figure 5). Additionally, performing Dufrene-Legendre indicator species analysis showed that there were no *Actinobacteria* indicators for dead root communities in either watered or drought-stressed tissues. Collectively, these results suggest that their drought-driven shift is unlikely to be attributable to saprophytic activity stimulated through root death. Interestingly, in addition to the expected increase in *Actinobacteria* from watered to drought-stress observed within living roots, a small increase is also observable within the dead roots (Figure 5B). A cell viability assay (Supplementary Figure S9) demonstrated that a portion of cells within detached roots are still viable after 3 days, suggesting that overall detached roots might be continuing to function metabolically for a period of time, which could explain the observed slight *Actinobacterial* enrichment if plant metabolism is a primary driver of this phenomenon.

### Drought Enrichment of Actinobacteria Is Consistent Across Hosts and Drought Treatments

**FIGURE 6:**
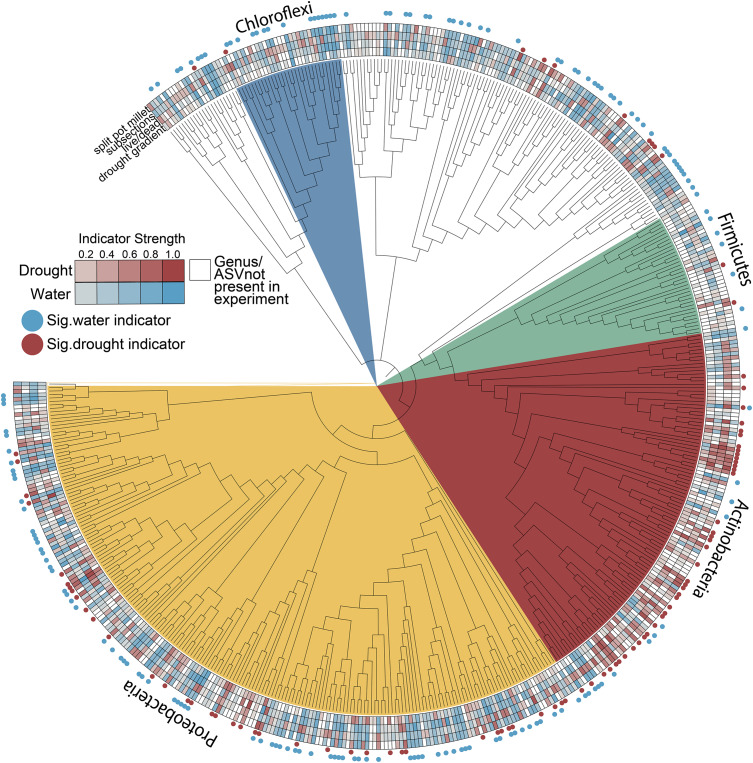
Root endophyte indicator values for drought and water conditions across all experiments. Nodes represent genera where sufficient taxonomic information was available to group ASVs by this rank; ASVs without genus-level annotations were included but ungrouped. Heatmaps connote indicator strength for drought and watered conditions (red and blue, respectively; white indicates taxa not present in a given experiment) and filled circles connote taxa that were a significant indicator of drought (red) or watered (blue) conditions in at least one experiment (*p* < 0.05, indcls >0.5).

We consistently observed enrichment of *Actinobacteria* across multiple experiments including both field and greenhouse studies, multiple millet species, and varying degrees and localizations of drought stress (Figure 6). While *Actinobacteria* as a phylum appears to become generally enriched under drought, other phyla such as the *Proteobacteria* are less consistent in their drought enrichment patterns, with taxa capable of being a significant indicator of both water and drought conditions across different experiments (Figure 6 and Supplementary Table S1). Interestingly, other phyla known to be composed of predominantly monoderm taxa such as the *Chloroflexi* and *Firmicutes* are not enriched under drought and in fact show more significant indicators of watered conditions (Figure 6).

## Discussion

### Actinobacterial Enrichment Under Drought Occurs Irrespective of Root Tissue Age

Our study provides an increased-resolution spatial dissection of the effect of drought stress on the development of the root microbiome and addresses several hypotheses regarding the underlying causes of recently reported increases in *Actinobacteria* that accompany drought stress. Through an exploration of the microbial communities in whole root systems, single roots, and sub-sectioned roots, we found an enrichment of *Actinobacteria* is a common phenomenon along the apical axis of a root (Figure 3). Since the majority of endophytic colonization of the root is thought to occur at the root tip and at positions where lateral roots are emerging (Shyam et al., 2017), this suggests that the underlying cause of enrichment is not simply increased rates of colonization by *Actinobacteria*, but perhaps also increased proliferation of established *Actinobacterial* endophytes within older root tissue, in comparison to other bacterial phyla. If correct, this implies that any plant-derived molecular signal that contributes to this phenomenon should be present not only within rhizosphere exudates, but also within the endosphere compartment as well. Several proposed molecular mechanisms for the observed *Actinobacterial* enrichment, including shifts in amino acids and carbohydrate biosynthesis and ROS production (Xu and Coleman-Derr, 2019), would likely affect both rhizosphere and endosphere compartments.

Other studies have explored how root associated microbial communities change across the apical axis of the root under non-drought conditions (Liljeroth et al., 1991; Yang and Crowley, 2000; Baudoin et al., 2002; Kawasaki et al., 2016). In a comparative analysis of microbiome composition between the root tip and root base of nodal roots in *Brachypodium*, Kawasaki et al. (2016) identified a relative increase in relative abundance of both *Betaproteobacteria* and *Gammaproteobacteria* lineages within the younger, growing root tip as compared to the root base. Interestingly, our data do not display a similar pattern of enrichment for these lineages, which suggests bacterial taxa may have preferential colonization rates at the root tip that differ across hosts or environments.

### Actinobacterial Enrichment Under Drought Is Localized to Sites of Drought Application

While it remains unclear what host mechanisms underlie the cause of the increase in *Actinobacteria* within the root system under drought, our data demonstrate that this enrichment is observed only within roots that are experiencing drought, and not found across the entire root system. For this reason, we propose that host-mediated causes would lie in localized host responses to drought, rather than systemic responses. This would, for instance, potentially exclude shifts in plant metabolites synthesized in the leaves and transported into the root system, that likely result from altered rates of photosynthesis during drought (Pinheiro and Chaves, 2011).

Shifts in plant metabolism during drought that are localized to portions of roots subjected to drought have been identified. For example, it was recently shown that in soils with heterogeneous moisture levels, there is an increased accumulation of abscisic acid (ABA), the phytohormone regulator of drought stress response, within roots found in drier regions of soil as compared to those found in regions of higher moisture (Puértolas et al., 2015). The effect of plant-produced ABA on the root microbiome has yet to be determined, though ABA is known to turn on genes for ROS production in the apoplast (Miller et al., 2010; Brito et al., 2019), which could have an impact on the bacterial community (Xu and Coleman-Derr, 2019). Perhaps more importantly, ABA acts antagonistically to systemic levels and activity of salicylic acid (SA) (de Torres Zabala et al., 2009) and in turn SA has been shown to influence root microbiome composition (Lebeis et al., 2015; Liu et al., 2018). Additionally, it is interesting to note that *Actinobacteria*, such as *Streptomyces*, are known to trigger systemic acquired resistance (SAR), traditionally associated with pathogens (Newitt et al., 2019). Taken together, this suggests that the enrichment of *Actinobacteria* may be driven by a localized hormone mediated response to drought, and that this enrichment itself may drive additional systemic changes in plant immunity.

### Actinobacterial Enrichment Under Drought Is Not Driven by Root Death

Many soil *Actinobacteria* function as saprophytes, consuming dead organic material (Barka et al., 2016). We had hypothesized that *Actinobacteria* may perceive root death within the drought-stressed root system and that this triggers their increased activity and abundance. However, we demonstrate that microbial communities of severed roots had fewer *Actinobacteria* than intact roots under both watered and drought treatments; in fact, *Actinobacteria* are the predominant indicators of living roots tissue. It is possible saprophytic colonization and activity does contribute to long term *Actinobacterial* increases under drought, and that such shifts take longer to develop than the time frame used in this study.

While historically categorized as free-living saprophytes, recent work on the root microbiome suggests that many *Actinobacteria* may have a less well understood phase of development or lifestyle associated with the endosphere, which leads to alternate functions and potentially even changes in cellular morphology (Ramijan et al., 2018; van der Meij et al., 2018). It is known that some bacteria occupy different niches (i.e., play different functional roles) depending on the presence of certain environmental triggers, such as carbon sources (Duffy and Défago, 1999; Sánchez et al., 2010). Indeed, some *Actinobacterial* lineages long considered saprophytic have been shown to, under certain environmental conditions, enhance plant growth through competition with plant pathogens (Millard and Taylor, 1927; Newitt et al., 2019). Since it is unknown what triggers the switch to a saprophytic lifestyle, and *Actinobacteria* are abundant in both living and dead roots, we could hypothesize that the bacteria are attracted to inert components of plant cell walls that are present under both conditions, and the endophytes do not express saprophytic functions within the living root environment.

### Variation in Enriched Genera Within Actinobacteria

Drought-induced enrichment of *Actinobacteria* has been observed in this study across multiple experiments with different host plants, which supports a growing body of evidence that this is a widespread pattern during drought (Naylor et al., 2017; Santos-Medellín et al., 2017; Edwards et al., 2018; Fitzpatrick et al., 2018; Xu et al., 2018). Additionally, our work supports previous studies that show differences in enrichment at finer taxonomic resolution (Naylor et al., 2017; Fitzpatrick et al., 2018). That is, though *Actinobacteria* show consistent enrichment as a phylum, the families and genera that are enriched may vary between host plants or experiments (Figure 6). *Streptomyces* is perhaps the most notable *Actinobacteria* genus that has been described to have plant-growth promoting abilities, particularly during abiotic or pathogen stress (Yandigeri et al., 2012; Qin et al., 2015; Singh et al., 2016; Xu et al., 2018; Newitt et al., 2019). While *Streptomyces* are known to produce spores, previous studies have ruled out spore-production as the sole explanation for *Actinobacterial* enrichment under drought as there are other enriched *Actinobacterial* genera that do not contain the genetic prerequisites for spore formation (Naylor et al., 2017). Additional dissection of the host and microbial molecular response to drought stress using a combination of genetic and omic tools may help to narrow down the underlying cause of this phenomenon.

## Conclusion

It has been well established that the composition of the root microbiome varies based on host genetics (Naylor et al., 2017; Fitzpatrick et al., 2018), host age (Edwards et al., 2018; Xu et al., 2018), environment (Bulgarelli et al., 2012; Lundberg et al., 2012; Edwards et al., 2015), and proximity to the root (Naylor and Coleman-Derr, 2018). Recent studies have demonstrated that during drought stress, there is an enrichment of *Actinobacteria* in the root endosphere, and this occurs across taxonomically diverse plant hosts (Naylor et al., 2017; Santos-Medellín et al., 2017; Edwards et al., 2018; Fitzpatrick et al., 2018; Xu et al., 2018). In this study, we used drought-tolerant millets to investigate where in the root system this enrichment occurs, in order to better understand the driving force behind it. We first show that location along the root apical axis does not affect this enrichment, suggesting that the signal is not specific to one root-zone. Subsequently, we demonstrate that the enrichment occurs only in roots that are directly perceiving drought, therefore it is not likely due to a signal that moves throughout the root system. Finally, we show that a specific localized response–root death–is not the primary cause of *Actinobacteria* enrichment. Future efforts to identify the underlying molecular causes of this phenomena are clearly necessary, and the results presented here may help inform such efforts.

## Data Availability Statement

The datasets generated for this study can be found in the NCBI Short Read Archive at accession PRJNA607579, https://github.com/colemanderr-lab/Simmons-2020.

## Author Contributions

PB and AH designed the multi-species field experiment. TS and DC-D designed the greenhouse, lab, and sorghum field experiments. AG and RP performed sample collection and phenotypic measurements for the multi-species experiment. TS performed the sample collection for the remaining experiments, sample preparation, and library preparation. GP performed the microscopy. TS, AS, and DC-D performed the statistical analyses and manuscript preparation.

## Conflict of Interest

The authors declare that the research was conducted in the absence of any commercial or financial relationships that could be construed as a potential conflict of interest.
