# Supplementary material for: Drought Drives Spatial Variation in the Millet Root Microbiome
Source: Front Plant Sci. 2020 May 28;11:599. doi: 10.3389/fpls.2020.00599 (PMC7270290; doi:10.3389/fpls.2020.00599)
Supplement: Supplementary file 1 [file Data_Sheet_1.pdf]

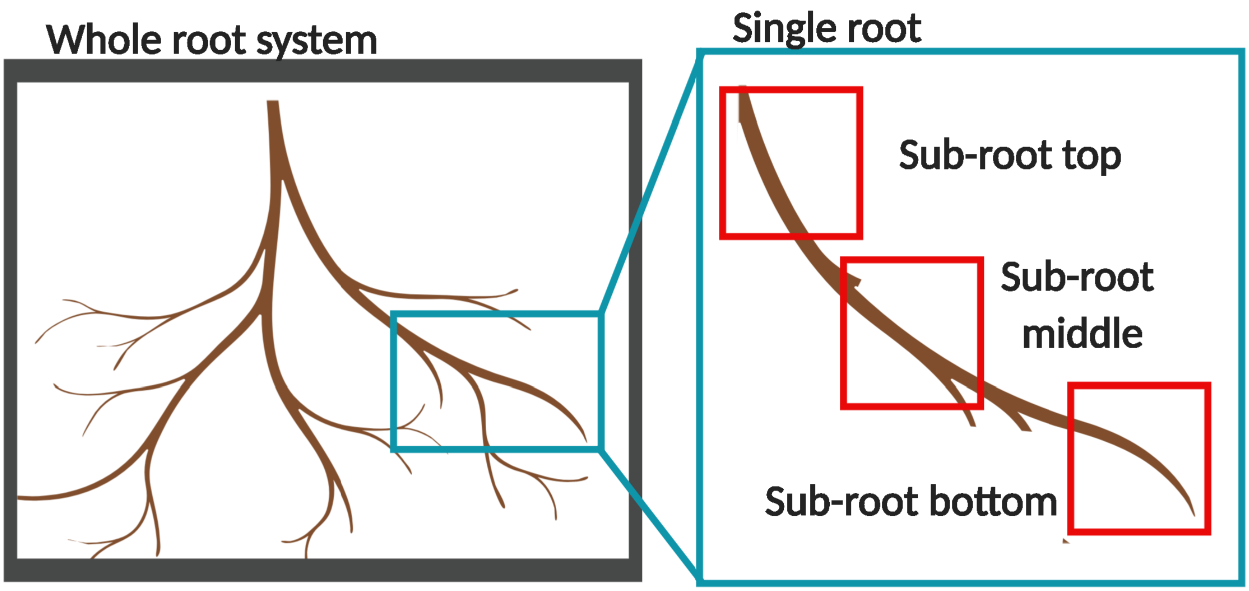

Figure S1.
Diagram showing sampling method of roots and root subsections from sorghum roots. After the plant was harvested, six individual roots were separated (three for Single Root samples, and three to be sub-sectioned), and the remaining roots were pooled together. Created with BioRender.

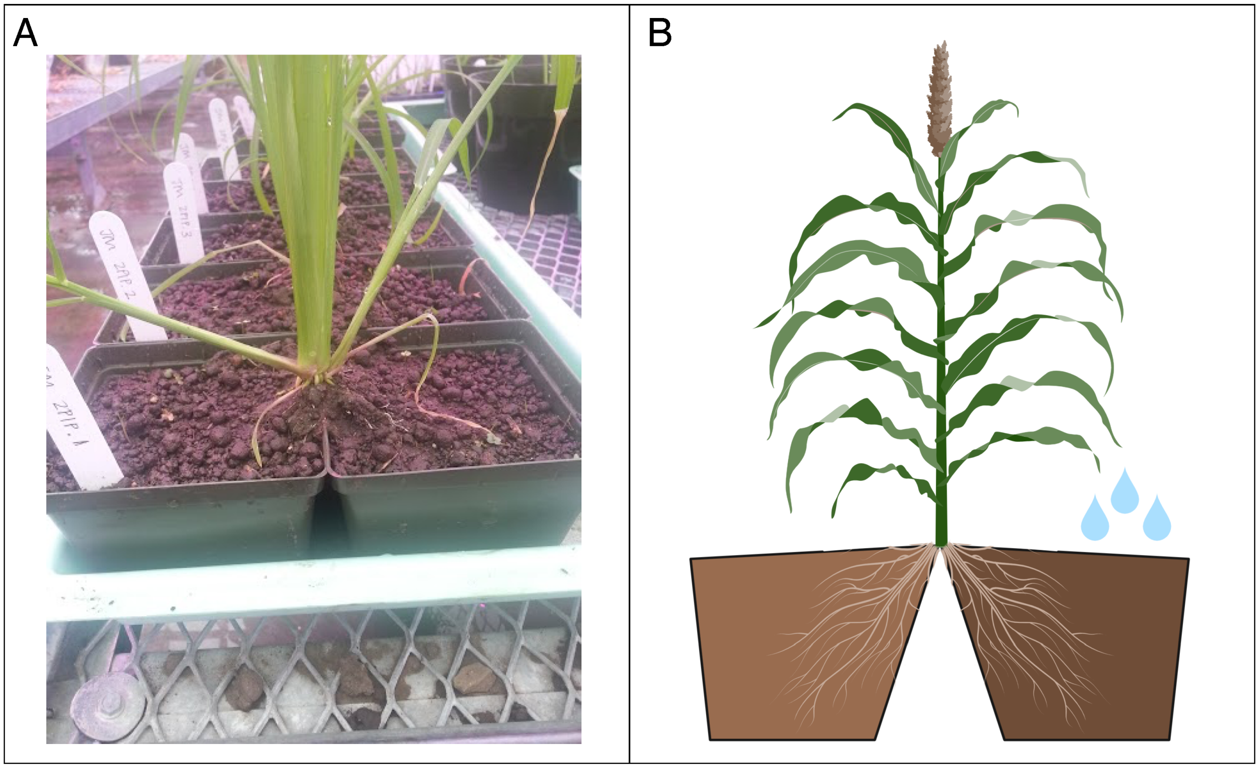

Figure S2.
Image taken from greenhouse set-up of Japanese millet growing in a split-pot system (A) and a diagram showing the watering regime for the split-pot treatment (B). Created with BioRender.

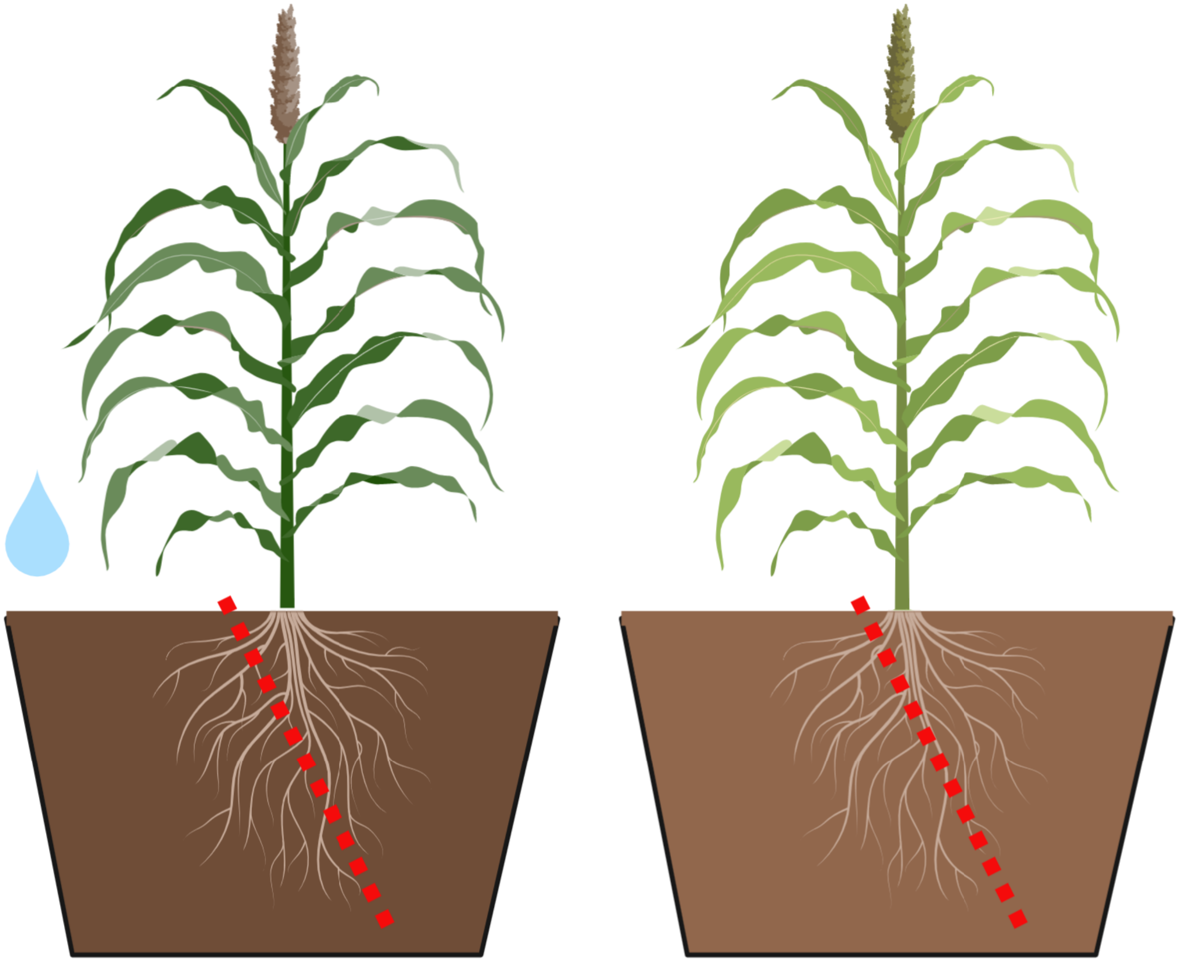

Figure S3.
Diagram showing set-up for live-dead root experiment. Blade was pushed through the roots approximately along the dotted red line, and half the plants were subjected to drought stress. Created with BioRender.

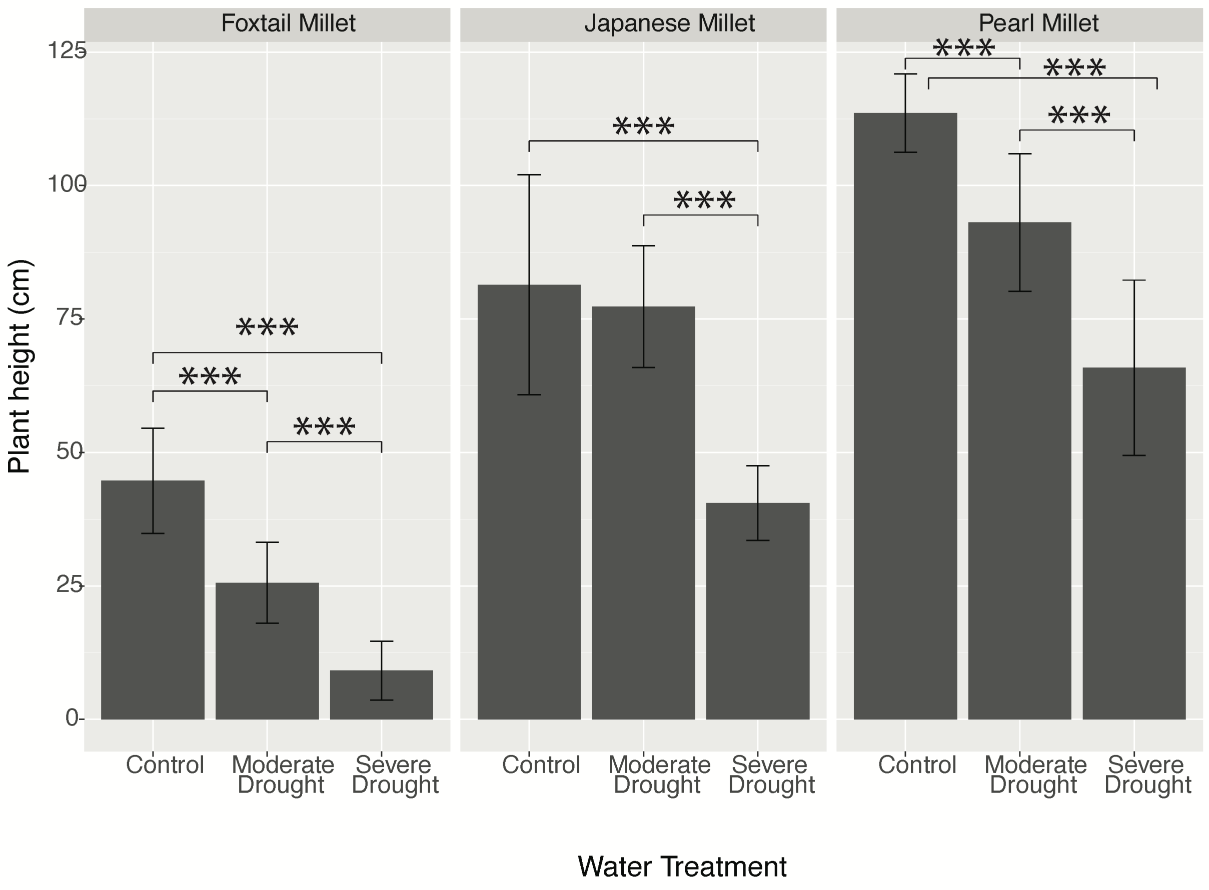

Figure S4.
Phenotypic data from multi-species millet field experiment. Plant height in centimeters (n=25). Vertical lines show standard deviation, and asterisks indicate statistical significance (*=p<0.05, **=p<0.01, ***=p<0.0001) according to the Wilcoxon rank sum test. Data is not available for proso millet due to destruction by native fauna.

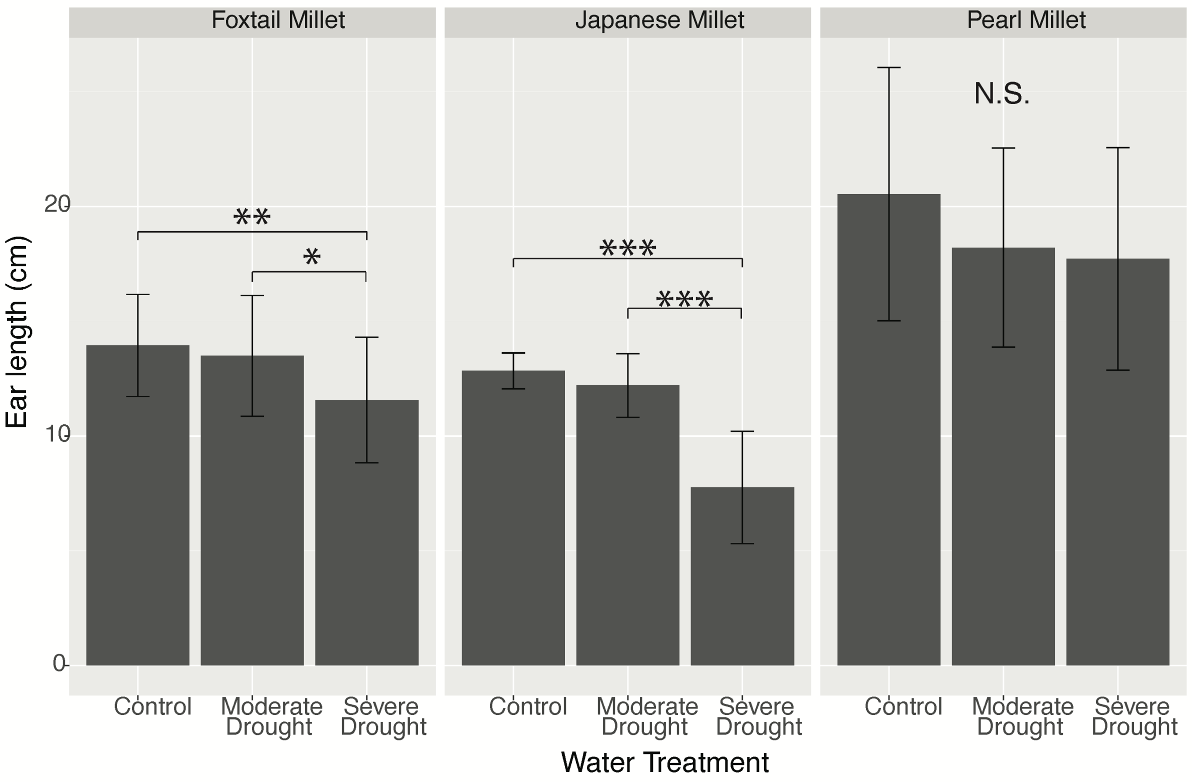

Figure S5.
Phenotypic data from multi-species millet field experiment. Ear length in centimeters (n=25). Vertical lines show standard deviation, and asterisks indicate statistical significance (*=p<0.05, **=p<0.01, ***=p<0.0001, N.S.=no significance) according to the Wilcoxon rank sum test. Data is not available for proso millet due to destruction by native fauna.

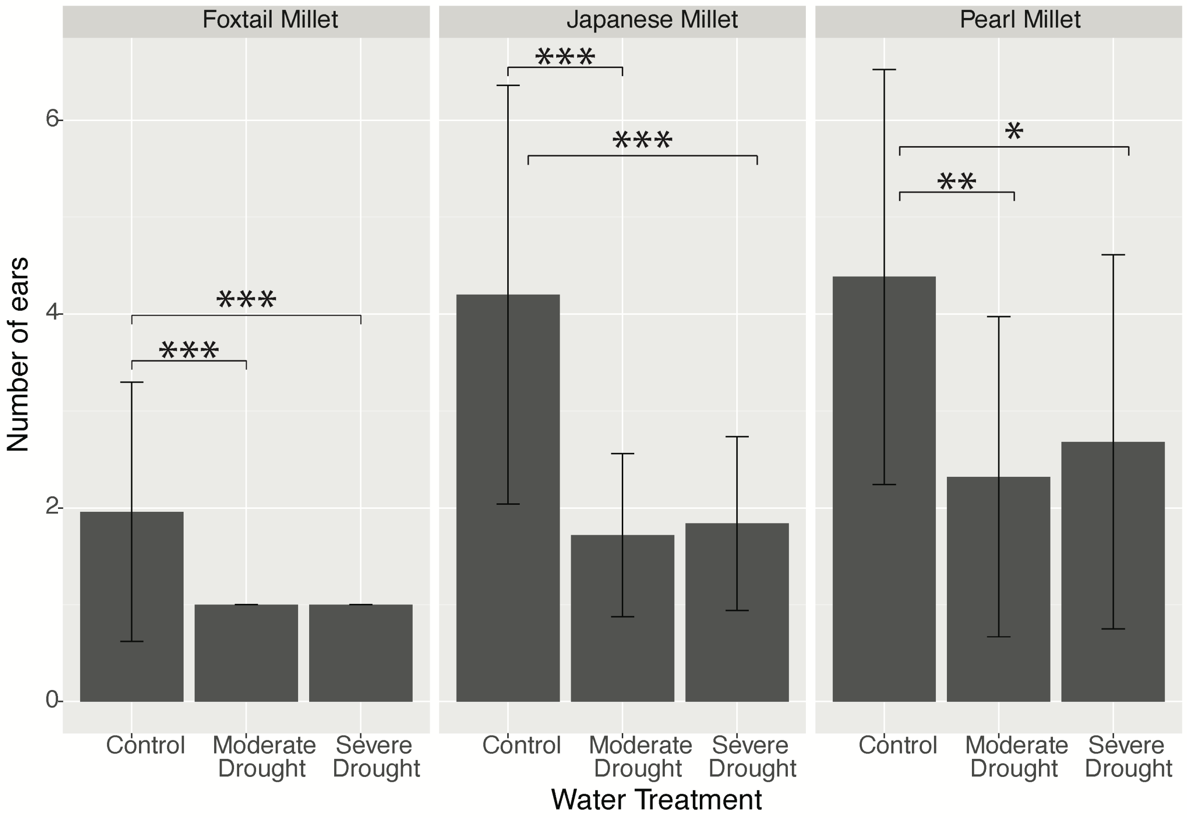

Figure S6.
Phenotypic data from multi-species millet field experiment. Number of ears per plant (n=25 except Pearl Millet Control, n=23). Vertical lines show standard deviation, and asterisks indicate statistical significance (*=p<0.05, **=p<0.01, ***=p<0.0001) according to the Wilcoxon rank sum test. Data is not available for proso millet due to destruction by native fauna.

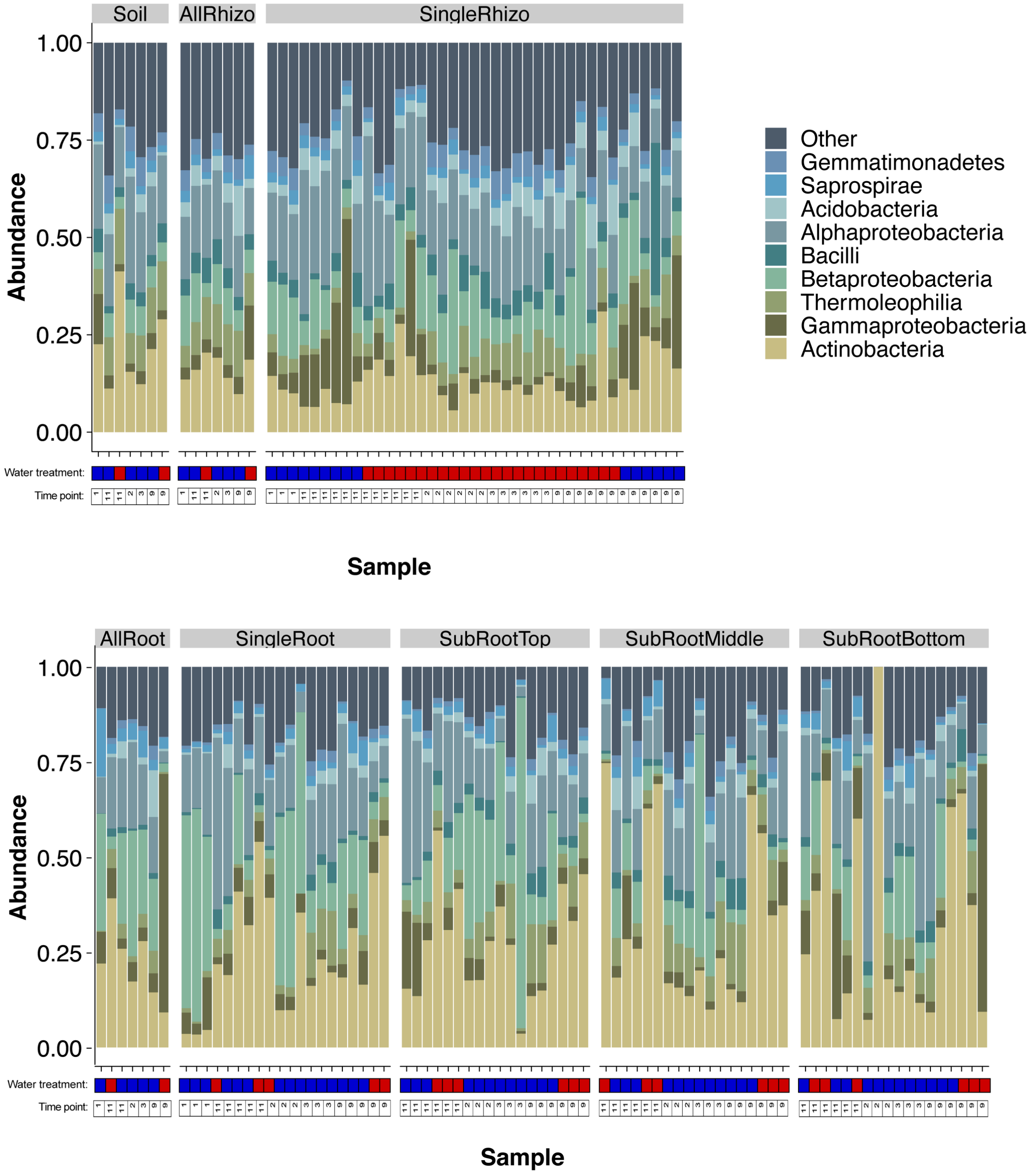

Figure S7.
Relative abundance of the top 9 most abundant bacterial classes within all samples in the subsection experiment. Each column represents a single sample. Colored blocks below columns indicate whether the sample was under well-watered (blue) or drought (red) conditions. Numbers under colored blocks represent the time point the sample was collected (in weeks).

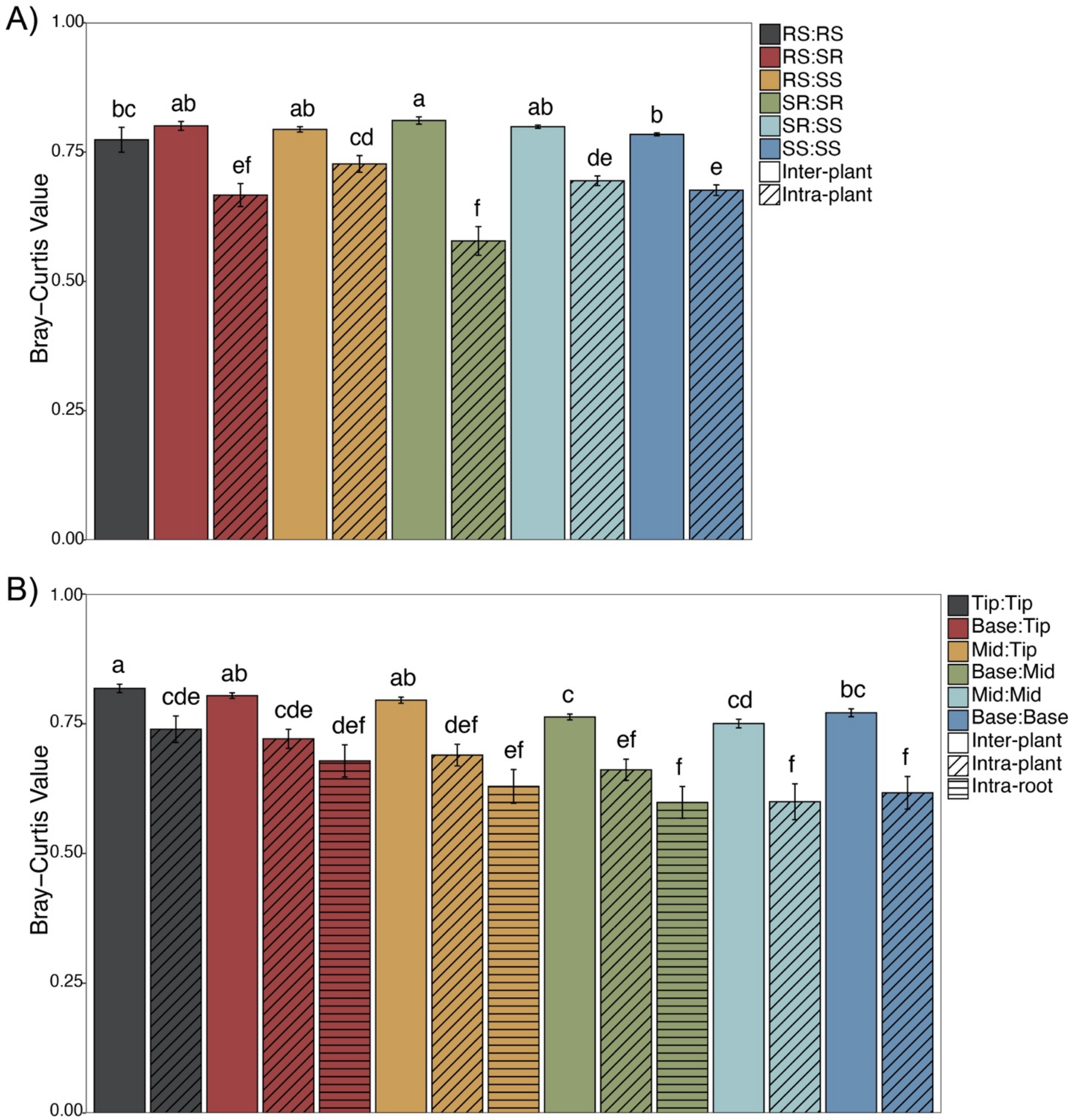

Figure S8.
Bray-Curtis dissimilarities between different sample types: RS – whole root system, SR – single roots, SS – subsection (A); in (B) all samples are subsections further classified as root tips, root middles, and root bases. In both (A) and (B), no matter the sample type, comparisons between samples of the same plant are more similar compared to samples from different plants. (B) additionally shows that samples from the same root are more similar compared to samples from different roots from either the same or different plants.

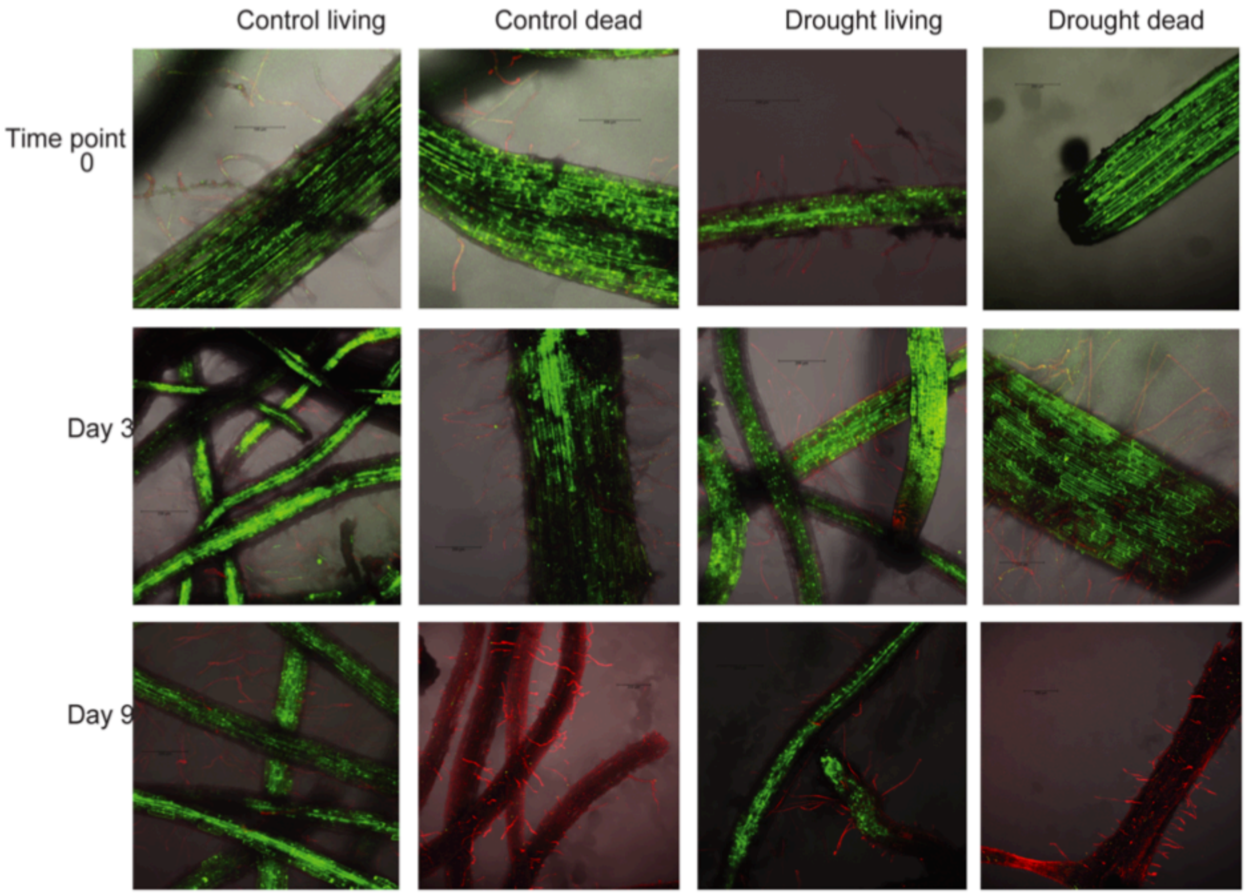

Figure S9.
Fluorescence microscopy of roots in live-dead experiment using Sigma-Aldrich's Plant Cell Viability Assay kit. Green indicates intact cell membranes and red indicates ruptured cell membranes. Assay was performed on living or detached roots in either drought or control water conditions on the day of detachment (time point 0), and 3 and 9 days post-detachment.

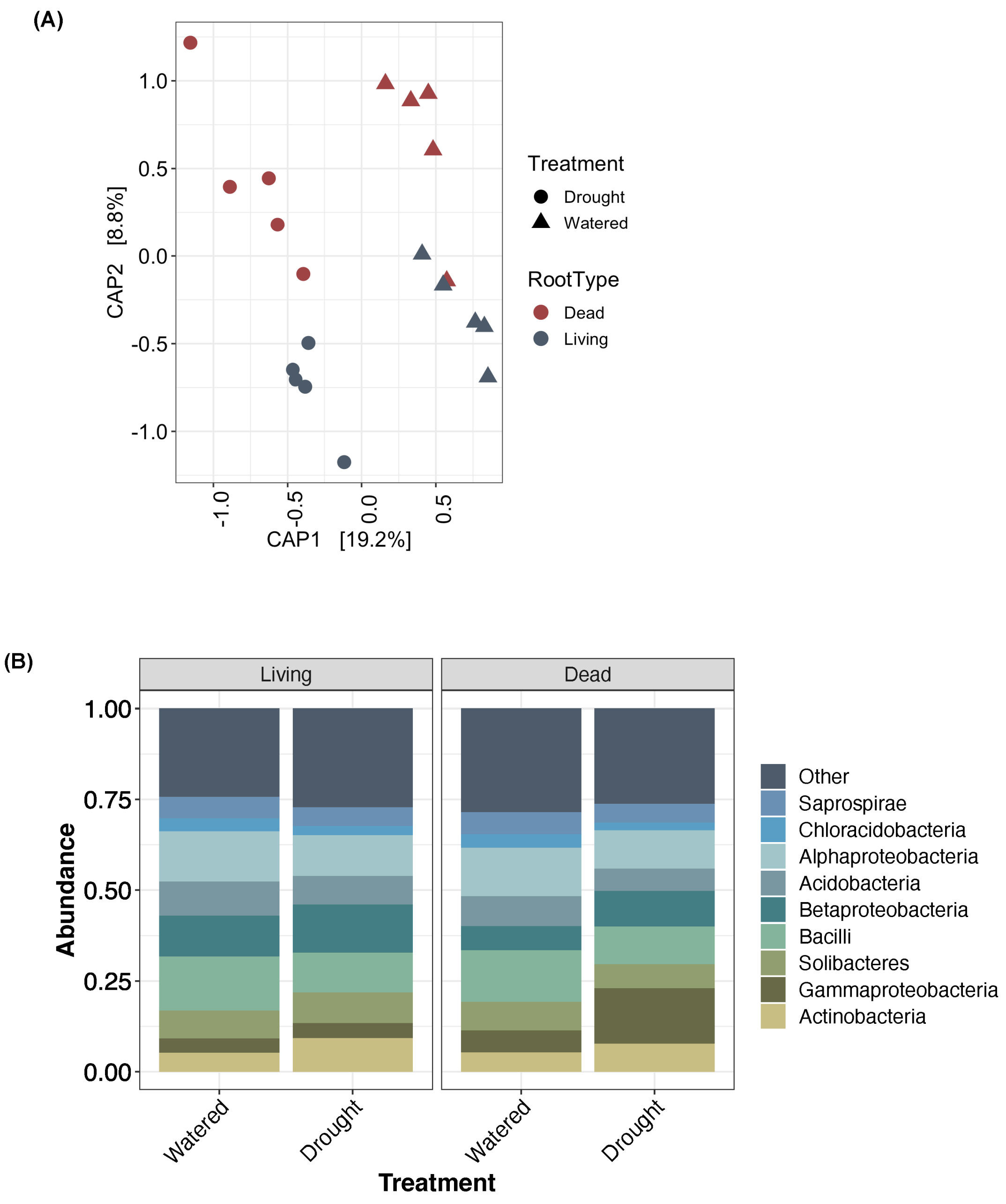

Figure S10.
Impact of root death on Japanese millet rhizosphere communities. A) CAP plot of root endosphere samples colored by root type and shaped by water treatment. B) Relative abundance of the top 9 most abundant bacterial classes in the root endosphere separated by water treatment.

Table S1.
Numbers of indicators for drought and water conditions across all experiments tabulated phylum. For this analysis ASVs were grouped by genera where taxonomic information was available at this level; nodes, then, represent both merged genera and ASVs unannotated at the genus level. These are the top 10 phyla by total number of nodes.

	# Drought Indicators	# Water Indicators	Drought/ Water	Total nodes representing phyla
Acidobacteria	1	10	0.1	23
Actinobacteria	40	14	2.86	110
Bacteroidetes	7	18	0.39	41
Chloroflexi	1	12	0.08	27
Cyanobacteria	0	2	0	5
Firmicutes	2	6	0.33	35
Gemmatimonadetes	0	6	0	8
Proteobacteria	38	74	0.51	201
TM7	1	1	1	8
Verrucomicrobia	0	9	0	20
